# Knowledge and Perceived Effectiveness of Infection Prevention and Control Measures Among Health Care Workers During the COVID-19 Pandemic: A National Survey

**DOI:** 10.1097/NCQ.0000000000000615

**Published:** 2021-12-20

**Authors:** Muna Abed Alah, Sami Abdeen, Nagah Selim, Dhouha Hamdani, Eman Radwan, Nahla Sharaf, Huda Al-katheeri, Iheb Bougmiza

**Affiliations:** Community Medicine Department, Hamad Medical Corporation (HMC), Doha, Qatar (Drs Abed Alah and Abdeen); Public Health and Preventive Medicine, Cairo University, Egypt (Dr Selim); Department of Family and Community Medicine (Dr Selim) and Community Medicine Department (Dr Bougmiza), Primary Health Care Corporation (PHCC), Doha, Qatar; Health Care Quality Management and Patient Safety Department (Ms Hamdani and Drs Radwan and Sharaf) and Strategic Planning and Performance Department (Ms Al-katheeri), Ministry of Public Health (MOPH), Doha, Qatar; and Community Medicine Department, College of Medicine, Sousse University, Tunisia (Dr Bougmiza).

**Keywords:** COVID-19, hand hygiene, health care worker, infection prevention and control, personal protective equipment, Qatar

## Abstract

Being the first line of defense against COVID-19 infection, health care workers (HCWs) are at an increased risk of getting infected. Infection prevention and control (IPC) measures were deemed to be instrumental in protecting them and their patients against infection.

**Purpose:**

To assess HCWs' knowledge of IPC measures and their perceived effectiveness in protecting against COVID-19.

**Methods:**

A national web-based survey was conducted in different health care sectors in Qatar.

**Results:**

A total of 1757 HCWs completed the survey. HCWs believed in applying stricter IPC precautions while dealing with confirmed COVID-19 cases than with suspected cases. Males and physicians were more likely to have high perceived effectiveness of IPC measures than females, nurses, and pharmacists. Higher proportions of HCWs believed in the effectiveness of hand hygiene than most types of personal protective equipment.

**Conclusion:**

Further research is recommended to assess the impact of HCWs' knowledge and perceived effectiveness of IPC measures on their compliance.

The rapid spread of coronavirus disease (COVID-19) worldwide overwhelmed health care capacities everywhere. Being the first line of defense against COVID-19 infection, health care workers (HCWs) are particularly at an increased risk of getting infected while dealing with increasing numbers of infected patients.^1^ More than 50% of HCWs got infected with SARS-CoV-2 in several countries according to a recently published systematic review and meta-analysis.^1^ The review included 28 studies involving 119 883 patients and showed that 51.7% of HCWs in the included studies collectively tested positive for COVID-19, with a hospitalization rate of about 15% and a mortality rate of 1.5%.^1^ In Qatar, a recent study has shown that 10.6% of HCWs tested positive for COVID-19, with 11.6% of them hospitalized.^2^ It is important for HCWs to comply with standard precautions such as the proper use of personal protective equipment (PPE), proper hand hygiene, and respiratory hygiene practices to contain the spread of the infection in health care facilities.

The World Health Organization (WHO) recommends HCWs follow droplet and contact precautions (medical mask, eye protection [goggles] or facial protection [face shield], nonsterile long-sleeved gown, and gloves) while caring for suspected or confirmed COVID-19 patients and airborne precautions using an N95 respirator or equivalent in addition to contact precautions while performing an aerosol-generating procedure (AGP). It also recommends practicing regular hand hygiene.^3^ However, such protective measures are of no benefit if used incorrectly due to lack of knowledge or used inconsistently by HCWs who do not believe in their effectiveness in preventing the spread of the infection. Evidence has shown that higher perceived effectiveness of PPE predicted higher compliance.^4^,^5^ The incorrect or inconsistent use of infection prevention and control (IPC) measures can result in health care–associated infections leading to prolonged hospital stays, massive additional costs for health systems and organizations, and unnecessary deaths.^6^–^9^

To the best of our knowledge, studies assessing HCWs' knowledge of proper IPC measures to be followed during COVID-19 and their perceived effectiveness during this pandemic are limited, particularly in the Middle East. This study aimed to assess HCWs' knowledge of the appropriate use of PPE and hand hygiene practices in different health care sectors in Qatar (governmental, semigovernmental, and private sectors) during the COVID-19 pandemic and to assess HCWs' perception of the effectiveness of different IPC measures in protecting against COVID-19 infection.

## METHODS

### Study design, setting, and target population

A web-based cross-sectional survey targeting the clinical staff (physicians, nurses, dentists, pharmacists, and allied health professionals) was conducted between November 2020 and January 2021. In Qatar, health care services are provided by governmental, semigovernmental, and private health care sectors. All HCWs in these sectors were invited to complete the survey. The governmental health care sector provides most of the health care services to the population of Qatar through Primary Health Care Corporation (PHCC), which provides primary health care services through different health centers distributed all over the country, and Hamad Medical Corporation, which provides secondary and tertiary care with several designated hospitals. The private sector includes more than 40 private hospitals and clinics. The semigovernmental sector includes 6 health care facilities.

### Ethical considerations

An ethical approval was obtained from the institutional review boards of relevant health institutions.

### Study procedure

A web-based self-administered survey was developed using Microsoft Forms software. All eligible HCWs in PHCC (as a major part of the governmental sector), semigovernmental, and private facilities were invited to take the survey to overcome the issue of low response rates usually encountered in web-based surveys. The link to the electronic version of the questionnaire was sent to HCWs via email. The survey started with an introductory letter that explained the objectives of the study and assured voluntary participation, anonymity, and confidentiality of the collected data. Taking the survey implied informed consent, and the participants had the option to quit the survey at any time. Reminders were sent regularly on a weekly basis.

### Overview of questionnaire

The questionnaire was adopted from other surveys in English.^10^–^13^ The face and content validity of the questionnaire was assured by infection control specialists. To examine the face validity, 2 infection control specialists independently evaluated the questionnaire for feasibility, readability, consistency of style, formatting, and clarity of the language used. They used a dichotomous scale with categorical options of “Yes” (favorable item) and “No” (unfavorable item) to evaluate the different items of the questionnaire. Their evaluation results were analyzed and showed satisfactory interrater agreement. To examine content validity, an additional infection control specialist and 2 community medicine specialists reviewed the questionnaire and rated the items as follows: 1, not relevant; 2, somewhat relevant; 3, quite relevant; and 4, highly relevant. Their results were evaluated by calculating a content validity index and showed satisfactory content validity. The internal consistency of the questionnaire using the Cronbach α was found to be 0.85.

The final questionnaire consisted of 3 sections (see Supplemental Digital Content, Data Collection Tool, available at: http://links.lww.com/JNCQ/A936). The first section consisted of 10 items that addressed the sociodemographic characteristics of the participants (age, gender, nationality, profession, clinical experience, health care facility), in addition to general information such as being aware of a friend or a relative infected with COVID-19, PPE and hand hygiene training, and frequency of dealing with suspected or confirmed COVID-19 cases. The second section consisted of 5 items that assessed HCWs' knowledge of appropriate types of PPE to be used in different settings and of hand hygiene practices. The third section consisted of 7 items that assessed HCWs' perception of the effectiveness of different IPC measures (PPE and hand hygiene) in protecting against COVID-19 infection.

### Study outcomes

HCWs' knowledge of the appropriate use of IPC measures was assessed in the second section of the questionnaire by asking participants about the types of PPE items that should be used in 3 different situations including regular patient interactions while dealing with suspected COVID-19 cases or confirmed cases, or while performing an AGP for suspected or confirmed case. They had the option to select more than 1 PPE item for each situation including (medical mask, N95 respirator or equivalent, eye protection [goggles] or facial protection [face shield], long-sleeved gown, and gloves). Answers were presented as percentages of participants selecting each PPE item. Participants were asked to indicate their degree of agreement with 2 statements on a 4-point Likert scale to assess their hand hygiene knowledge. These statements were as follows: “No need to practice hand hygiene if I'm wearing gloves” and “Hand washing using water and soap is as effective as using alcohol-based hand rub for preventing transmission of COVID-19.” HCWs' perceived effectiveness of IPC measures in protecting against COVID-19 was assessed in the third section of the questionnaire by asking them to indicate their degree of agreement with a set of statements on a similar 4-point Likert scale from 1 (strongly disagree) to 4 (strongly agree). Examples of these statements are as follows: “I believe that a regular face mask (medical or surgical) is effective and can help against contracting COVID-19 infection”; “I believe that an N95 respirator or equivalent is effective and can help against contracting COVID-19 infection”; and “Hand hygiene is effective and can help in preventing COVID-19 transmission.” Also, they were asked to indicate the degree of protection IPC measures can provide on a 5-point Likert scale. The points on the scale were 1 (no or very low protection), 2 (low protection), 3 (moderate protection), 4 (high protection), and 5 (very high protection). A perceived effectiveness score was calculated by summing the points for all statements. The maximum score was 29. Higher scores indicated higher perceived effectiveness of IPC measures in protecting against contracting COVID-19.

### Statistical analysis

Data analysis was performed using the IBM SPSS Statistics for Windows, version 26 (IBM Corp, Armonk, New York). Descriptive statistics were presented as frequencies and percentages for categorical variables. A chi-square test was used to determine the differences between categorical variables. Multivariable logistic regression was executed to determine the predictors of high perceived effectiveness of IPC measures. The associations between the predictors and the outcomes were presented as adjusted odds ratios (ORs) and 95% CIs. Goodness of fit was assessed using the Hosmer-Lemeshow test. *P* values of less than .05 were considered significant.

## RESULTS

### Sociodemographic characteristics and general information

A total of 1757 HCWs completed the survey. The majority (n = 757; 43.1%, were from the governmental sector (PHCC), 520 (29.6%) from the private sector, and 480 (27.3%) were from the semigovernmental sector. Almost half (49.9%) were between 30 and 39 years of age, and 1192 (67.8%) were females. Of more than 60 nationalities reported, most common were Filipino (29.8%) and Indian (27.4%). Only 32 (1.8%) participating HCWs were Qatari nationals. The top 3 professions reported were nurses, who accounted for the majority of HCWs (47.5%), allied health professionals (22%), and physicians (20.1%). The Figure describes the sociodemographic characteristics of the participating nurses. Of the participants, 1573 (89.5%) reported 5 or more years of clinical experience. More than 90% of the HCWs reported receiving training for proper PPE use and hand hygiene practices. More than one-third (35.2%) of the HCWs reported frequent interactions with suspected or confirmed COVID-19 cases (every work shift or most of their work shifts). Of all participating HCWs, 1354 (77.1%) had a relative, friend, or colleague diagnosed with COVID-19 (see Supplemental Digital Content, Table, available at: http://links.lww.com/JNCQ/A937).

**Figure.**
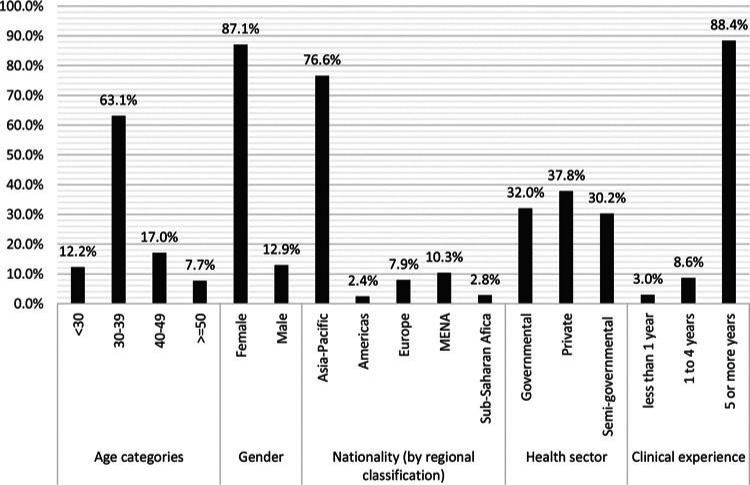
Sociodemographic characteristics of the nurses (represented as percentages of the total number of participating nurses). MENA indicates Middle East and North Africa.

### HCWs' knowledge of the appropriate types of IPC measures to use

On assessing HCWs' knowledge of the appropriate types of PPE to be used in different settings, 1284 (73.1%) reported that full-contact precautions (the combination of eye protection, long-sleeved gown, and gloves) must be used while dealing with suspected COVID-19 cases, 1559 (88.7%) while dealing with confirmed cases, and 1525 (86.8%) while performing an AGP for suspected or confirmed COVID-19 cases. About one-third of the participants (n = 533; 30.3%) reported that droplet precautions (using a regular face mask) but not airborne precautions (using an N95 respirator or equivalent) must be used when dealing with suspected cases, and 100 (5.7%) reported it when dealing with confirmed cases. On the contrary, 1211 (68.9%) and 1649 (93.9%) participants reported that a respirator must be used instead in these cases, respectively. The majority (n = 1672; 95.2%) indicated that respirators but not face masks must be used while performing an AGP for suspected or confirmed cases. Most of HCWs (86.1%) reported the need to practice hand hygiene even when wearing gloves, and 87.3% considered hand washing using water and soap as effective as using alcohol-based hand rub for preventing transmission of COVID-19.

### HCWs' perception of the effectiveness of different IPC measures against COVID-19 infection

On assessing HCWs' perceived effectiveness of IPC measures in protecting against contracting COVID-19 infection, the study showed that the majority of HCWs agreed or strongly agreed that a regular face mask (medical or surgical) (86.5%), a respirator (98.5%), eye protection (using goggles or face shield) (96.0%), long-sleeved gown (94.5%), and gloves (94.1%) were effective in protecting against COVID-19 infection. Most (97.8%) of the participants perceived hand hygiene as an effective IPC measure against COVID-19. Of the participants, 43.1% believed that IPC measures provide high protection and 46.4% believed very high protection against contracting the infection. The median perceived effectiveness score was 26 (IQR = 23-28). Of all HCWs, 924 (52.6%) scored 26 or more. As shown in the Table, significantly higher proportions of HCWs with high perceived effectiveness were found among males than among females (*P* < .001), among those who received PPE training (*P* = .014) and hand hygiene training (*P* = .047) in the previous year compared with those who did not, and among those who had a relative or friend diagnosed with COVID-19 compared with those who did not (*P* = .023). Nationality and profession were significantly associated with the perceived effectiveness (*P* < .001).

### Predictors of the perceived effectiveness of IPC measures

A multivariable logistic regression model was executed to determine the predictors of the perceived effectiveness of IPC measures, taking into consideration the perceived effectiveness score as high (≥26) and low (<26) as a dependent binary variable. Selection of the independent variables to be included in the model was based on clinical and statistical relevance. The model was statistically significant when compared with the null model (*P* < .001) and of good fit according to the Hosmer-Lemeshow test (*P* = .22). Gender, nationality, profession, and having a relative, friend, or colleague diagnosed with COVID-19 were significantly and independently associated with the perceived effectiveness. Males were found to be more likely to have high perceived effectiveness of IPC measures than females (adjusted OR = 1.59; 95% CI, 1.26-2.00; *P* < .001). HCWs who were aware of a friend, relative, or colleague diagnosed with COVID-19 were also more likely to have high perceived effectiveness than those who were not (adjusted OR = 1.31; 95% CI, 1.04-1.65; *P* = .023). On the contrary, HCWs with nationalities of European (adjusted OR = 0.52; 95% CI, 0.36-0.75; *P* < .001) and Middle Eastern-North African (adjusted OR = 0.49; 95% CI, 0.37-0.65; *P* < .001) origins were less likely to have high perceived effectiveness than those of Asian-Pacific origins. Nurses (adjusted OR = 0.64; 95% CI, 0.47-0.88; *P* = .007) and pharmacists (adjusted OR = 0.46; 95% CI, 0.30-0.73; *P* = .001) were less likely to report high perceived effectiveness than physicians. However, no significant associations were found between each of health sector, clinical experience, and previous IPC training and the outcome (Table).

**Table.** Determinants and Predictors of Perceived Effectiveness of IPC Measures Using the Chi Square Test and Multiple Logistic Regression Analysis

	Perceived Effectiveness of IPC Measures^a^				
				Multivariable Regression Analysis	
Variables	Low Perceived IPC Effectiveness, n (%)	High Perceived IPC Effectiveness, n (%)	χ^2^ Test *P* Value^b^	AOR (95% CI)	*P* b
Age categories					
<30 y	91 (48.4)	97 (51.6)	.123	1 [Reference]	
30-39 y	411 (46.9)	465 (53.1)		0.83 (0.59-1.18)	.303
40-49 y	233 (51.0)	224 (49.0)		0.68 (0.46-1.03)	.066
≥50 y	98 (41.5)	138 (58.5)		0.96 (0.61-1.51)	.846
Gender					
Female	606 (50.8)	586 (49.2)	**<.001**	1 [Reference]	
Male	227 (40.2)	338 (59.8)		1.59 (1.26-2.00)	**<.001**
Nationality (by regional classification)					
Asia-Pacific	455 (42.9)	605 (57.1)	**<.001**	1 [Reference]	
Americas	25 (39.7)	38 (60.3)		0.99 (0.56-1.75)	.967
Europe	111 (54.1)	94 (45.9)		0.52 (0.36-0.75)	**<.001**
MENA	193 (56.8)	147 (43.2)		0.49 (0.37-0.65)	**<.001**
Sub-Saharan Africa	49 (55.1)	40 (44.9)		0.67 (0.42-1.07)	.096
Profession					
Allied health professional	171 (44.3)	215 (55.7)	**<.001**	0.75 (0.53-1.05)	.091
Dentist	16 (32.0)	34 (68.0)		1.46 (0.76-2.83)	.251
Nurse	412 (49.4)	422 (50.6)		0.64 (0.47-0.88)	**.007**
Pharmacist	84 (62.7)	50 (37.3)		0.46 (0.30-0.73)	**.001**
Physician	150 (42.5)	203 (57.5)		1 [Reference]	
Health sector					
Governmental	353 (46.6)	404 (53.4)	.192	1 [Reference]	
Private	236 (45.4)	284 (54.6)		0.92 (0.71-1.18)	.485
Semigovernmental	244 (50.8)	236 (60.0)		0.86 (0.66-1.12)	.262
Clinical experience					
<1 y	12 (40.0)	18 (46.1)	.182	1 [Reference]	
1-4 y	83 (53.9)	71 (46.1)		0.58 (0.25-1.32)	.194
≥5 y	738 (46.9)	835 (53.1)		0.75 (0.35-1.62)	.467
Aware of a relative, friend, or colleague diagnosed with COVID-19					
No	211 (52.4)	192 (47.6)	**.023**	1 [Reference]	
Yes	622 (45.9)	732 (54.1)		1.31 (1.04-1.65)	**.023**
Appropriate PPE use training in the past year					
No	87 (56.9)	66 (43.1)	**.014**	1 [Reference]	
Yes	746 (46.5)	858 (53.5)		1.41 (0.94-2.13)	.101
Appropriate hand hygiene training in the past year					
No	45 (58.4)	32 (41.6)	**.047**	1 [Reference]	
Yes	788 (46.9)	892 (53.1)		1.13 (0.65-1.97)	.654

Abbreviations: AOR, adjusted odds ratio; IPC, infection prevention and control; MENA, Middle East and North Africa; PPE, personal protective equipment.

^a^Participants were divided into 2 categories using the median of perceived effectiveness score as a cutoff point (<26 low perceived IPC effectiveness, ≥26 high perceived IPC effectiveness).

^b^P values of less than .05 were considered significant and are shown in bold.

## DISCUSSION

The spread of COVID-19 infection placed HCWs among the highly exposed groups. They can serve as a potential source of infection to others by transmitting the virus between patients to other people in their working environment and even to their families and friends at home. Their compliance with IPC measures is critically important to protect themselves and others against the spread of infectious diseases. This study assessed HCWs' knowledge of the proper use of IPC measures and their perceived effectiveness in protecting against the spread of COVID-19.

The WHO, the Centers for Disease Control and Prevention (CDC), and national IPC guidelines in Qatar do not differentiate between dealing with suspected or confirmed cases in their IPC-related recommendations. However, our study showed differences in HCWs' knowledge of the types of precautions to be used while dealing with confirmed or suspected cases. They reported the need of stricter precautions when dealing with confirmed cases than with suspected cases. Small proportions (30.3% and 5.7%) of HCWs seemed to base their knowledge on the WHO's recommendations, as they reported that droplet but not airborne precautions using a face mask must be used during regular patient interactions with suspected or confirmed cases, respectively, which can be explained by the fact that the national IPC guidelines in Qatar recommends the use of a respirator instead of a face mask in such cases consistent with the CDC's recommendations.^14^ However, the CDC also recommends the use of a face mask if a respirator is not available.^14^ We believe that HCWs' knowledge reflects their actual practice, so clarifying this point to HCWs is needed considering the global shortage of PPE to optimize their rational use.

More than 95% of the participants knew that an N95 respirator or equivalent must be used while performing an AGP; all international (WHO, CDC) and national recommendations agree on that point. More than 80% of the participants knew that it is essential to practice hand hygiene even when wearing gloves and that hand washing using water and soap is as effective as using alcohol-based hand rub for preventing transmission of COVID-19 similar to what was reported in a study in Pakistan^15^ and higher than what was reported in Ethiopia.^16^

Collectively, 89.5% of the HCWs believed that IPC measures provide high to very high protection against contracting COVID-19. However, the data related to HCWs' perceived effectiveness of different IPC measures indicate that HCWs believe that some IPC measures are superior to others in preventing the spread of infection, particularly when it comes to face masks and respirators as 86.5% and 98.5% of the HCWs found them effective, respectively. This result is similar to what was reported in a study in Singapore during severe acute respiratory syndrome (SARS).^10^ This study showed that 97.8% of the HCWs perceived hand hygiene as an effective IPC measure against COVID-19, which is higher than what was found in a recently published study in Uganda, where 88% of HCWs believed this.^17^ Studies conducted during SARS epidemic have also shown that HCWs perceived hand washing as a highly effective measure against infection.^18^,^19^

Nationalities of European and Middle Eastern-North African origins were less likely to have high perceived effectiveness than those of Asian-Pacific origins. One explanation might be that Europe and the Middle East have gone through difficult experiences during this pandemic in terms of the number of cases, deaths, and the spread of infection, which might be attributed by many to ineffectiveness of IPC measures. Thus, HCWs of those origins might have been impacted unconsciously by the pandemic situations in their home countries. Nurses and pharmacists were found less likely to have high perceived effectiveness than physicians. This might be attributed to the stronger medical background physicians generally have than other HCWs, which might affect the way they perceive the effectiveness of IPC measures.

Although PPE comes at the bottom of the hierarchy of hazard controls at workplaces, it remains essential to protect HCWs and patients from the spread of infections, especially at challenging times such as with the COVID-19 pandemic when other measures such as through engineering and administrative controls are not sufficient to stop the spread of the virus. The correct choice of the type of PPE to be used is critically important in light of a global PPE shortage, as unnecessarily wearing more expensive PPE items such as N95 respirators when simple face masks can be used would put other HCWs in need for such PEE at risk of encountering infections. The low perceived effectiveness of IPC measures can lead to inconsistent use of such measures, which might trigger health care–associated infections. Health care organizations need to ensure that HCWs are aware of the correct combination of PPE to be used in different settings to help optimize their rational use. Further research is needed to explore the impact of the perceived effectiveness of IPC measures on HCWs' compliance with such measures. We believe that HCWs who have high perceived effectiveness of IPC measures would be more compliant and more confident in dealing with suspected or confirmed cases while wearing PPE, which eventually will improve their abilities to deliver high-quality care to their patients.

### Strengths and limitations

This is the first national study in Qatar to address HCWs' perceived effectiveness of IPC measures during the COVID-19 pandemic. We managed to enroll an acceptable number of HCWs from different health care sectors, strengthening the external validity of this study. While our study is among the limited literature that investigated the perceived IPC effectiveness, there are some noteworthy limitations that should be acknowledged. First, different individual institutional IPC recommendations might have affected the responses of HCWs. Second, hindered by the length of the survey, we limited the knowledge assessment to the appropriate types of PPE to be used in different settings and did not assess HCWs' knowledge of the donning and doffing mechanisms, and the correct steps of performing hand hygiene, although we believe that the assessment of these aspects would be better by direct observation rather than self-reporting. The inconsistent use of IPC measures by HCWs who do not believe in their effectiveness or who are unaware of the appropriate types of PPE to be used in different situations can potentiate the spread of infections in health care settings, which poses a risk to HCWs and patients.

## CONCLUSION

In this study, knowledge assessment results reflected that HCWs in Qatar believe in applying stricter IPC precautions when dealing with confirmed COVID-19 cases than dealing with suspected cases. More than 85% of the HCWs believed in the effectiveness of different IPC measures in protecting against the spread of COVID-19. Higher proportions of HCWs believed in the effectiveness of hand hygiene than most types of PPE. On assessing the perceived effectiveness score, more than 50% of HCWs scored high (≥26). This study showed that gender, nationality, profession, and having a relative, friend, or colleague diagnosed with COVID-19 were significantly and independently associated with perceived effectiveness.

## Supplementary Material

SUPPLEMENTARY MATERIAL
