# Supplementary material for: Knowledge and Perceived Effectiveness of Infection Prevention and Control Measures Among Health Care Workers During the COVID-19 Pandemic: A National Survey
Source: J Nurs Care Qual. 2021 Dec 20;37(2):E23–30. doi: 10.1097/NCQ.0000000000000615 (PMC8860130; doi:10.1097/NCQ.0000000000000615)
Supplement: SUPPLEMENTARY MATERIAL [file jncqu-37-e23-s001.docx]

Supplemental Digital Content. Data Collection Tool

| **Section A**: **Socio-demographic characteristics and general information:** | | | | | | | | | | | | | | | | | | | | | | | | | | |
| --- | --- | --- | --- | --- | --- | --- | --- | --- | --- | --- | --- | --- | --- | --- | --- | --- | --- | --- | --- | --- | --- | --- | --- | --- | --- | --- |
| **Question** | | | | **Choices:** | | | | | | | | | | | | | | | | | | | | | | |
| 1. Age | | | |  | | | | | | | | | | | | | | | | | | | | | | |
| 1. Gender | | | | Male | | | | | | | | | | | Female | | | | | | | | | | | |
| 1. Nationality | | | |  | | | | | | | | | | | | | | | | | | | | | | |
| 1. Profession | | | | Physician | | | | | Nurse | | pharmacist | | | | Dentist | | | | Allied Health | | | Others, specify | | | | |
| 1. Place of work/Facility name | | | |  | | | | | | | | | | | | | | | | | | | | | | |
| 1. Clinical experience | | | |  | | | | | | | | | | | | | | | | | | | | | | |
| 1. How frequently do you deal with COVID-19 suspected or confirmed cases during your clinical practice? | | | | Every shift | | | | | | Most of my shifts | | | | | | Some of my shifts | | | | | | | | | | never |
| 1. Are you aware of any relative or friend of yours who has been diagnosed with COVID-19 infection? | | | | Yes | | | | | | | | | | | | No | | | | | | | | | | |
| 1. Have you received any form of training regarding the appropriate use of PPE in the previous year? | | | | Yes | | | | | | | | | | | | No | | | | | | | | | | |
| 1. Have you received any form of training about the appropriate hand hygiene practices in the previous year? | | | | Yes | | | | | | | | | | | | No | | | | | | | | | | |
| **Section B**: **Knowledge of the appropriate use of infection prevention and control measures (IPC):** | | | | | | | | | | | | | | | | | | | | | | | | | | |
| 1. According to your knowledge what is/are the appropriate PPE that should be used when dealing with **suspected** COVID-19 cases? (**you can choose more than one answer**) | Regular Face mask (medical or surgical) | | | | respirator (N95, FFP2 or powered air purifying respirator (PAPR)) | | | | | | | eye protection (googles) or facial protection (face shield) | | | | | long sleeved gown | | | | | | Gloves | | | |
| 1. According to your knowledge what is/are the appropriate PPE that should be used when dealing with **Confirmed** COVID-19 cases? (**you can choose more than one answer**) | Regular Face mask (medical or surgical) | | | | respirator (N95, FFP2 or powered air purifying respirator (PAPR)) | | | | | | | eye protection (googles) or facial protection (face shield) | | | | | long sleeved gown | | | | | | gloves | | | |
| 1. According to your knowledge what is/are the appropriate PPE that should be used when dealing with suspected or confirmed COVID-19 cases during **aerosol generating procedure**? **(you can choose more than one answer**) | Regular Face mask (medical or surgical) | | | | respirator (N95, FFP2 or powered air purifying respirator (PAPR)) | | | | | | | eye protection (googles) or facial protection (face shield) | | | | | long sleeved gown | | | | | | gloves | | | |
| 1. No need to practice hand hygiene if I’m wearing gloves | Strongly agree | | | | | | | agree | | | | | | | | | | disagree | | | | | | strongly disagree | | |
| 1. Hand washing using water and soap is as effective as using alcohol-based hand rub for preventing transmission of COVID-19 | Strongly agree | | | | | | | agree | | | | | | | | | | disagree | | | | | | strongly disagree | | |
| **Section C**: **Perception of effectiveness infection prevention and control measures (IPC):** | | | | | | | | | | | | | | | | | | | | | | | | | | |
| 1. To what extent do you feel that infection prevention and control measures (PPE, hand hygiene and others) are protective against contracting COVID-19 infection? | | Very low protection | | | | Low protection | | | | | | | Moderate protection | | | | | | | High protection | | | | | Very high protection | |
| *Please indicate your degree of agreements with the following statements as it applies to the healthcare setting where you work:* | | | | | | | | | | | | | | | | | | | | | | | | | | |
| 1. I believe that regular face mask (medical or surgical) is effective and can help against contracting COVID-19 infection. | | | Strongly agree | | | | agree | | | | | | | disagree | | | | | | | strongly disagree | | | | | |
| 1. I believe that respirator (N95, FFP2 or PAPR) is effective and can help against contracting COVID-19 infection. | | | Strongly agree | | | | agree | | | | | | | disagree | | | | | | | strongly disagree | | | | | |
| 1. I believe that googles or facial shield are effective and can help against contracting COVID-19 infection. | | | Strongly agree | | | | agree | | | | | | | disagree | | | | | | | strongly disagree | | | | | |
| 1. I believe that long sleeve gown is effective and can help against contracting COVID-19 infection. | | | Strongly agree | | | | agree | | | | | | | disagree | | | | | | | strongly disagree | | | | | |
| 1. I believe that gloves are effective and can help against contracting COVID-19 infection. | | | Strongly agree | | | | agree | | | | | | | disagree | | | | | | | strongly disagree | | | | | |
