# Supplementary material for: Knowledge and Perceived Effectiveness of Infection Prevention and Control Measures Among Health Care Workers During the COVID-19 Pandemic: A National Survey
Source: J Nurs Care Qual. 2021 Dec 20;37(2):E23–30. doi: 10.1097/NCQ.0000000000000615 (PMC8860130; doi:10.1097/NCQ.0000000000000615)
Supplement: SUPPLEMENTARY MATERIAL [file jncqu-37-e23-s002.docx]

| Characteristic |  | N (%) |
| --- | --- | --- |
| Age categories | <30 | 188 (10.7) |
|  | 30-39 | 876 (49.9) |
|  | 40-49 | 457 (26.0) |
|  | >=50 | 236 (13.4) |
| Gender | Female | 1192 (67.8) |
|  | Male | 565 (32.2) |
| Nationality^a^ (by regional classification) | Asia-Pacific | 1060 (60.3) |
|  | Americas | 63 (3.6) |
|  | Europe | 205 (11.7) |
|  | Middle East-North Africa | 340 (19.4) |
|  | Sub-Saharan Africa | 89 (5.1) |
| Profession | Allied health professional | 386 (22.0) |
|  | Dentist | 50 (2.8) |
|  | Nurse | 834 (47.5) |
|  | Pharmacist | 134 (7.6) |
|  | Physician | 353 (20.1) |
| Health sector | Governmental | 757 (43.1) |
|  | Private | 520 (29.6) |
|  | Semi-Governmental | 480 (27.3) |
| Clinical experience | less than 1 year | 30 (1.7) |
|  | 1 to 4 years | 154 (8.8) |
|  | 5 or more years | 1573 (89.5) |
| Aware of a relative, friend or colleague diagnosed with COVID-19 | No | 403 (22.9) |
|  | Yes | 1354 (77.1) |
| Appropriate PPE use training in the past year | No | 153 (8.7) |
|  | Yes | 1604 (91.3) |
| Appropriate Hand Hygiene training in the past year | No | 77 (4.4) |
|  | Yes | 1680 (95.6) |

Supplemental Digital Content Table. Sociodemographic Profiles and Background Information of the Participants

^a^ More than 60 different nationalities were reported.
